# 20 years of blood management and VTE prevention in orthopedic surgery in the Netherlands – a nationwide survey

**DOI:** 10.1007/s00402-026-06301-8

**Published:** 2026-04-23

**Authors:** Ruben Y. Kok, Joost-Jelle T. M. Gerritsen, Cees C. P. M. Verheyen, Banne Németh, Harmen B. Ettema

**Affiliations:** 1 Department of Clinical Epidemiology, Leiden University Medical Center, Leiden, Netherlands; 2 Department of Orthopedics, Isala, Zwolle, Netherlands; 3 Department of Orthopaedics, Leiden University Medical Center, Leiden, Netherlands

**Keywords:** Venous thromboembolism, Thromboprophylaxis, Hip arthroplasty, Knee arthroplasty, Bleeding

## Abstract

**Introduction:**

Advances in arthroplasty care have progressively improved patient outcomes, including reduced bleeding and venous thromboembolism rates. Consequently, blood-saving measures applied pre-, peri- and postoperatively as well as thromboprophylaxis regimens have evolved over time. To evaluate these developments, we conducted a 2023 follow-up to the national surveys from 2002, 2007 and 2012, examining current blood-saving strategies and thromboprophylaxis use in The Netherlands.

**Materials and methods:**

In 2023, a questionnaire designed to match the previous surveys was distributed to all orthopedic departments in the Netherlands. The collected data were summarized and compared with previous findings.

**Results:**

Responses were received from 61% of the Dutch orthopedic departments. Blood-saving practices have shifted significantly toward the use of tranexamic acid, accompanied by a decline in other measures. In particular, the use of drains, blood transfusions, and erythropoietin supplementation has decreased substantially. Thromboprophylaxis protocols showed fewer changes, although the average duration of prophylaxis has shortened, with low-molecular-weight heparin remaining the most commonly used anticoagulant.

**Conclusions:**

Advances in surgical techniques and postoperative care around arthroplasty appear to have reduced concerns about perioperative blood loss to a level where the use of tranexamic acid alone is considered sufficient to control blood loss, rendering many previously popular perioperative blood-saving measures redundant. In contrast, despite a decline in thrombotic complications associated with the introduction of fast-track surgery, thromboprophylaxis protocols have remained largely unchanged. These findings highlight the opportunity to further balance the risks of bleeding and thrombosis following arthroplasty.

**Supplementary Information:**

The online version contains supplementary material available at 10.1007/s00402-026-06301-8.

## Introduction

Total hip and knee arthroplasty (THA/TKA) have traditionally been associated with substantial blood loss, necessitating various pre-, peri-, and postoperative blood management measures, including erythropoietin (EPO) supplementation, intra- or postoperative autologous (re)transfusion and drain placement. Effective blood management is essential, as wound hematoma is associated with wound complications such as persistent drainage or bleeding. Furthermore, both hematoma formation and blood transfusion have been associated with an increased risk of periprosthetic joint infection (PJI) [1, 2]. In contrast to blood loss, the risk for postoperative venous thromboembolism (VTE) has been high, necessitating (extended)thromboprophylaxis therapy [3–5]. 

Over the last 20 years, multiple surveys have been conducted among Dutch orthopedic surgeons to evaluate pre-, peri- and postoperative blood management and thromboprophylaxis measures in the Netherlands. The first two surveys (2002–2007), showed an increasing use of preoperative EPO and postoperative retransfusion of autologous blood [6]. In 2012, these trends continued: the vast majority of orthopedic clinics in the Netherlands used EPO and implemented peri- or postoperative blood transfusion [7]. At that time, tranexamic acid (TXA) was not yet widely adopted. In line with these anti-bleeding strategies, most anticoagulants were discontinued preoperatively. Thromboprophylactic therapy mostly consisted of extended prophylaxis with a low-molecular-weight-heparin (LMWH) up to 6 weeks following surgery [8, 9]. 

In recent decades arthroplasty care has changed drastically. The introduction of fast-track surgery, consisting of multimodal strategies such as minimal invasive procedures, optimal anesthesia and pain management to reduce surgical stress, has substantially improved perioperative course and outcomes [10]. Patients are mobilized immediately after surgery (rendering several blood-saving measures impractical) and hospitalization time has drastically reduced. With these advancements, VTE rates have declined to 1.0%−1.5% and blood loss has markedly decreased [11–13]. Since clinical practice changed considerably since the last survey, a new survey was performed to assess and to compare blood management and VTE prevention measures following arthroplasty in the Netherlands.

## Methods

In 2023, a questionnaire on orthopedic departmental protocols for blood management and thromboprophylaxis was sent to all orthopedic clinics in the Netherlands. A 3-month window was provided to complete the survey, non-respondents were sent multiple reminders to complete the survey. The questionnaire was designed to align with previous surveys on blood management in 2002, 2007 and 2012 and on thromboprophylaxis in 2002 and 2007. The survey included questions on pre-, peri- and postoperative blood management strategies, (dis)continuation of anticoagulants and use of thromboprophylaxis. Data collection was performed between March 2023 and October 2023. Survey questions were presented to orthopedic surgeons in a multiple-choice format. For each type of thromboprophylaxis and blood-saving intervention, surgeons indicated whether the intervention was applied in clinical practice and whether this was done routinely for the following procedures: total hip arthroplasty (THA), revision hip arthroplasty (RHA), hemi hip arthroplasty (HHA), total knee arthroplasty (TKA), revision knee arthroplasty (RKA), unicondylar knee arthroplasty (UKA).

For thromboprophylaxis, surgeons were additionally asked to specify the routinely prescribed duration of prophylaxis. With regard to perioperative management of anticoagulants, surgeons reported the number of days each type of anticoagulant was discontinued prior to surgery. Completion of all questions was required. The full survey is available in the supplementary data. (supplement 4)

Data from the current survey were compared with previous surveys to assess changes in clinical practice. Categorical and dichotomous variables were summarized as percentages with a corresponding Wilson 95% confidence interval (CI) using R version 4.5.0 (R Foundation for Statistical Computing, Vienna, Austria).

## Results

### Respondents demographics

A total of 48 out of 79 (61%) orthopedic departments in the Netherlands completed the survey. Respondents included 7 of 7 university hospitals, 16 of 18 teaching hospitals, 20 of 43 general hospitals and 5 of 11 independent treatment centers (ITCs). The characteristics of responding and non-responding orthopedic departments are shown in Table 1. Responding departments were more frequently academic (15%) and teaching hospitals (33%), whereas non-responding departments were predominantly general hospitals (74%). The geographical distribution was broadly comparable, although departments from the western region were more represented among non-responders (55%) compared with responders (42%). Responding departments tended to perform higher numbers of arthroplasty procedures annually, particularly revision surgeries, aligning with the higher percentage of academic and teaching hospitals. In addition, responding departments had a larger number of orthopedic surgeons across subspecialties. The availability of a departmental blood-saving protocol was reported by 95% of departments and all departments reported having a thromboprophylaxis protocol.

**Table 1:** Characteristics of responders and non-responders

		Responding departments	Non-responding departments
Hospital type (n; %)	Academic hospital	7 (15%)	0 (0%)
	Teaching hospital	16 (33%)	2 (6%)
	General hospital	20 (42%)	23 (74%)
	ITC	5 (10%)	6 (19%)
Region (n; %)	North	4 (8%)	5 (16%)
	East	12 (25%)	4 (13%)
	South	12 (25%)	5 (16%)
	West	20 (42%)	17 (55%)
Yearly surgeries (median; IQR)	THA	397 (IQR 225–675)	325 (IQR 190–563)
	RHA	52 (IQR 21–85)	26 (IQR 11–38)
	TKA	296 (IQR 211–464)	264 (IQR 203–401)
	RKA	37 (IQR 19–56)	20 (IQR 12–31)
	UKA	69 (IQR 28–134)	79 (IQR 38–115)
Orthopedic surgeons performing: (median; IQR)	Hip arthroplasty	6 (IQR 5–9)	4 (IQR 3.5–6)
	Revision hip	4 (IQR 2–6)	2 (IQR 2–4)
	Knee arthroplasty	6 (IQR 5–8)	4 (IQR 3.5–5.5)
	Revision knee	3 (IQR 3–5)	2 (IQR 2–4)

*IQR * Interquartile range, *ITC * Independent treatment centre, *THA *Total hip arthroplasty, *RHA *Revision hip arthroplasty, *TKA* Total knee arthroplasty, *RKA * Revision knee arthroplasty, *UKA *Unicondylar knee arthroplasty

### Preoperative blood-saving measures

**Table 2:** Preoperative blood-saving measures

	Autologous blood donation				Erythropoietin supplementation				Iron supplementation (oral or IV)	
	2002	2007	2012	2023	2002	2007	2012	2023	2012	2023
THA	14%	11%	4%	0% (CI 0% − 8%)	32%	65%	60%	17% (CI 9% − 31%)	29%	11% (CI 5% − 23%)
RHA	8%	10%	4%	2% (CI 0% − 11%)	31%	67%	57%	17% (CI 9% − 31%)	29%	11% (CI 5% − 23%)
HHA*	NA	NA	NA	0% (CI 0% − 8%)	NA	NA	NA	11% (CI 5% − 23%)	NA	8% (CI 3% − 19%)
TKA	9%	9%	4%	0% (CI 0% − 8%)	32%	64%	55%	17% (CI 9% − 31%)	26%	11% (CI 5% − 23%)
RKA	5%	9%	4%	0% (CI 0% − 8%)	25%	62%	57%	17% (CI 9% − 31%)	27%	11% (CI 5% − 23%)
UKA	3%	7%	2%	0% (CI 0% − 8%)	13%	41%	47%	11% (CI 5% − 23%)	23%	11% (CI 5% − 23%)

CIs represent 95% confidence intervals

*THA * Total hip arthroplasty, *RHA * Revision hip arthroplasty, *HHA * Hemi hip arthroplasty, *TKA * Total knee arthroplasty, *RKA * Revision knee arthroplasty, *UKA * Unicondylar knee arthroplasty.

*No data available prior to 2023

Consistent with previous trends, the use of autologous blood donation further declined to nearly 0%. Similarly, the use of EPO supplementation decreased from 47% to 60% in 2012 to 11%−17% in 2023. Over the same period the use of iron supplementation halved. No substantial differences were observed between types of surgery. An overview of preoperative blood-saving measures is presented in Table 2.

Preoperative discontinuation rates of different anticoagulants are shown in Table 3. Compared to 2012, non-steroidal anti-inflammatory drugs (NSAIDs), Cyclooxygenase-2 (COX 2) inhibitors, acetylsalicylic acid and dipyridamole are less frequently discontinued. In contrast, the discontinuation rate of clopidogrel increased to 92%. When discontinued, clopidogrel was usually stopped 5–7 days preoperatively, 68% of departments substituted clopidogrel with acetylsalicylic acid during the pre- and perioperative period. Vitamin K antagonists (VKAs) and direct oral anticoagulants (DOACs) were discontinued by all departments, with 85% of departments discontinuing DOACs 2 days preoperative.

**Table 3:** Preoperative discontinuation of anticoagulants

	2007	2012	2023
NSAID	41%	36%	10% (CI 4% − 22%)
COX 2 inhibitors	NA*	40%	7% (CI 2% − 18%)
Acetylsalicylic acid	83%	54%	11% (CI 3% − 22%)
Clopidogrel	NA*	84%	92% (CI 81% − 97%)
Dipyridamole	NA*	70%	48% (CI 33% − 60%)
Vitamin K antagonists	100%	98%	100% (CI 92% − 100%)
DOACs	NA*	NA*	100% (CI 92% − 100%)

CIs represent 95% confidence intervals

*NSAID* Non-steroidal anti-inflammatory drug, *COX 2 inhibitors* Cyclooxygenase-2 inhibitors, *DOACs* Direct oral anticoagulants

*Data were not collected in previous surveys

### Perioperative blood-saving measures

Tranexamic acid (TXA) is prescribed by 88%−97% of the departments; a substantial increase compared to 2012 (8%−15%). Among these departments, 84% administer TXA intravenously, 2% locally and 14% both locally and intravenously. In 92% TXA is administered within one hour prior to surgery. Intraoperative retransfusion of autologous blood (by means of a Cell Saver) decreased further for THA, RHA and RKA compared to 2012. Tourniquet use also declined considerably in knee arthroplasty from 85% to 90% in 2012 to 29%−41% in 2023 (Table 4). The use of normothermia, epinephrine, normovolemic hemodilution and controlled hypotension did not change since 2012. Fibrine glue use in THA is reported by only 4% of departments, and neither fibrin nor platelet gel are in current use. Complete results are available as supplementary Table 1.

**Table 4:** Perioperative blood-saving measures

	Intraoperative retransfusion				TXA (IV or local)		Tourniquet	
	2002	2007	2012	2023	2012	2023	2012	2023
THA	15%	19%	14%	7% (CI 2% − 18%)	14%	97% (CI 87% − 99%)	NA	NA
RHA	40%	54%	56%	15% (CI 8% − 28%)	15%	95% (CI 84% − 98%)	NA	NA
HHA	6%	12%	7%	7% (CI 2% − 18%)	8%	88% (CI 76% − 95%)	NA	NA
TKA	10%	14%	9%	7% (CI 2% − 18%)	12%	97% (CI 87% − 99%)	90%	38% (CI 25% − 52%)
RKA	16%	24%	26%	8% (CI 3% − 19%)	13%	95% (CI 84% − 98%)	87%	29% (CI 18% − 44%)
UKA	6%	11%	8%	7% (CI 2% − 18%)	9%	95% (CI 84% − 98%)	85%	41% (CI 28% − 56%)

CIs represent 95% confidence intervals

*THA * Total hip arthroplasty, *RHA * Revision hip arthroplasty, *HHA * Hemi hip arthroplasty, *TKA * Total knee arthroplasty, *RKA * Revision knee arthroplasty, *UKA * Unicondylar knee arthroplasty, *TXA *Tranexamic acid

### Postoperative blood-saving measures

Since 2012, both postoperative autologous blood retransfusion and the use of surgical drains have almost disappeared from clinical practice. Whereas drains were used in 72%–81% of arthroplasties in 2012, they are no longer used in hip arthroplasty, and only 1 department reports their use in knee arthroplasty. The use of cryotherapy in both hip and knee arthroplasty has also declined, whereas the use of a compression bandage has decreased only in hip arthroplasty. Leg elevation advise following surgery remains unchanged (Tables 5 and 6).

**Table 5:** Postoperative blood-saving measures

	Postoperative autologous retransfusion				Drain	
	2002	2007	2012	2023	2012	2023
THA	14%	58%	58%	2% (CI 0% − 11%)	72%	0% (CI 0% − 8%)
RHA	13%	54%	53%	4% (CI 1% − 15%)	79%	0% (CI 0% − 8%)
HHA	8%	41%	47%	2% (CI 0% −11%)	72%	0% (CI 0% − 8%)
TKA	23%	70%	65%	4% (CI 1% − 15%)	75%	2% (CI 0% − 11%)
RKA	15%	59%	61%	5% (CI 2% − 16%)	81%	2% (CI 0% − 11%)
UKA	8%	48%	53%	4% (CI 1% − 15%)	71%	2% (CI 0% − 11%)

CIs represent 95% confidence intervals

*THA* Total hip arthroplasty, *RHA* Revision hip arthroplasty, *HHA* Hemi hip arthroplasty, *TKA* Total knee arthroplasty, *RKA* Revision knee arthroplasty, *UKA * Unicondylar knee arthroplasty

*Data were not collected in previous surveys

**Table 6:** Postoperative blood-saving measures

	Compression bandage		Cryotherapy		Leg elevation	
	2012	2023	2012	2023	2012	2023
THA	30%	12% (CI 5% − 24%)	4%	2% (CI 0% − 11%)	NA*	7% (CI 2% − 18%)
RHA	30%	14% (CI 7% − 27%)	5%	0% (CI 0% − 8%)	NA*	7% (CI 2% − 18%)
HHA	26%	12% (CI 5% − 24%)	3%	0% (CI 0% − 8%)	NA*	7% (CI 2% − 18%)
TKA	63%	76% (CI 62% − 86%)	16%	12% (CI 5% − 24%)	17%	15% (CI 8% − 28%)
RKA	62%	68% (CI 54% − 80%)	12%	8% (CI 3% − 19%)	14%	13% (CI 6% − 26%)
UKA	60%	74% (CI 60% − 84%)	15%	8% (CI 3% − 19%)	17%	13% (CI 6% − 26%)

CIs represent 95% confidence intervals

*THA * Total hip arthroplasty, *RHA * Revision hip arthroplasty, *HHA *Hemi hip arthroplasty, *TKA * Total knee arthroplasty, *RKA * Revision knee arthroplasty, *UKA * Unicondylar knee arthroplasty

*Data were not collected in previous surveys

### Thromboprophylaxis strategies

Multiple types of pharmacologic thromboprophylaxis are used, LMWH remains the most commonly prescribed type of anticoagulation (Table 7). DOACs are prescribed more often following primary arthroplasty than following revision surgery. Unlike 2002 and 2007, VKAs and Fondaparinux are no longer in use as thromboprophylaxis. The use of LMWH remained stable since 2007 (supplementary Table 2).

**Table 7:** Type of pharmacological thromboprophylaxis

2023	LMWH	DOAC	LMWH + DOAC	DOAC + aspirin
THA	71%	21%	6%	1%
RHA	75%	17%	7%	1%
HHA	83%	9%	7%	1%
TKA	73%	21%	6%	1%
RKA	77%	16%	7%	1%
UKA	71%	21%	7%	1%

(Percentages may not total 100% due to rounding)

*LMWH* Low-molecular-weight heparin, *DOAC *Direct oral anticoagulant, *THA *Total hip arthroplasty, *RHA * Revision hip arthroplasty, *HHA* Hemi hip arthroplasty, *TKA *Total knee arthroplasty, *RKA* Revision knee arthroplasty, *UKA *Unicondylar knee arthroplasty

Among departments using a combined LMWH and DOAC strategy, 66% switched to a DOAC after discharge and 33% switched to a DOAC 1 week following surgery (total duration 4 weeks). One department reported a hybrid strategy with 5 days of DOAC therapy followed by 25 days of aspirin. In 10% of departments the choice between LMWH or DOAC depends on patient related factors (for example thrombocyte aggregation inhibitor (TAI) use or needle aversion). Of departments prescribing LMWH, 32% prescribe dalteparin and 68% nadroparin. Of departments prescribing a DOAC, 26% prescribe apixaban, 8% dabigatran and 66% rivaroxaban.

The mean duration of thromboprophylaxis decreased from 2002 to 2023. In 2023 prophylaxis duration ranged from 4 to 6 weeks in hip arthroplasty and 2–6 weeks in knee arthroplasty (Table 8). Overall, almost 50% of patients receive 4 weeks of thromboprophylaxis regardless of the type of arthroplasty. Detailed information on thromboprophylaxis duration in 2023 is provided in supplementary Table 3.

**Table 8:** Duration of the pharmacological thromboprophylaxis

	2002	2007	2023	
			Knee	Hip
In hospital*	3%	0%	0%	0%
< 4 weeks	0%	0%	12%	0%
4 weeks	0%	9%	48%	50%
> 4 weeks − 6 weeks	36%	85%	41%	50%
> 6 weeks	60%	5%	0%	0%

(Percentages may not total 100% due to rounding)

*Hospitalization after arthroplasty in 2002 was generally 10–14 days

## Discussion

### Summary of main findings

Current blood management practices in the Netherlands following hip and knee arthroplasty are mainly restricted to the use of TXA. Perioperative or postoperative blood transfusions (either autologous or allogeneic) are rarely performed. Only a small number of departments still administer preoperative pharmacological agents such as erythropoietin or iron. Hence, these results suggest that blood loss is no longer a major concern in arthroplasty. In line with these findings, most clinics continue aspirin and NSAIDs in the pre- and perioperative period.

Thromboprophylaxis is prescribed for approximately 4 weeks, with LMWH remaining the most commonly used agent in approximately 75% of departments, followed by DOACs in approximately 17%.

### Main differences with previous surveys

Previous surveys showed a strong increase in measures to decrease blood loss across pre-, peri- and postoperative strategies. From 2002 to 2007, the use of preoperative erythropoietin more than doubled from approximately 32% to 66% in hip arthroplasty and from 24% to 56% in knee arthroplasty, with similar rates reported in 2012. In contrast, data from 2023 showed a sharp decline to ~ 15% in both hip and knee replacement, with iron supplementation showing a similar decline. Perioperative measures such as autologous blood transfusion (e.g. Cell Saver) increased modestly between 2002 and 2012 but had declined by 2023. Likewise, tourniquet use in knee arthroplasty decreased by half over the same period.

Postoperatively, retransfusions of autologous blood increased between 2002 and 2012 (from 14% to 58% following hip arthroplasty and from 23% to 65% following knee arthroplasty, with a similar trend in revision surgery). By 2023, only 4% of clinics still perform postoperative retransfusions. The most widely adopted blood-saving measure in 2023 was TXA, now used in 95% of departments compared with only 12% in 2012. Altogether, these data suggest that most blood-saving strategies have been abandoned, except for TXA use.

In contrast to these major shifts in blood management, thromboprophylaxis strategies show relatively little changes since 2007. The average duration of thromboprophylaxis decreased from 6 to 4 weeks and LMWH remains the primary choice, while Fondaparinux (and VKA) have been phased out. DOACs, are now used in approximately 18% of arthroplasties.

### Guidelines

The findings of our study, which reflect current Dutch clinical practice, are largely consistent with national Dutch guidelines. The reduced use of preoperative measures can be explained by current guideline recommendations, which advise against routine use of autologous blood donation. In addition, the use of iron or erythropoietin supplementation is only advised for patients with preoperative anaemia undergoing high-risk procedures such as revision arthroplasty [14]. 

During the perioperative period, the Dutch anaesthesiology guideline recommends several measures including patient positioning to prevent venous congestion, maintaining normothermia and considering tourniquet use in all surgeries [14]. For procedures with an expected blood loss exceeding 40% of total blood volume, which is rare in arthroplasty, normovolemic haemodilution and controlled hypotension may be advised. Intraoperative retransfusion (Cell Saver) is similarly reserved for cases with substantial anticipated blood loss. Due to limited evidence, no specific recommendations are made regarding surgical haemostatic agents, except that fibrin glue may be used at the surgeon’s discretion. The guideline also supports the routine use of TXA in all arthroplasties.

The recent European Society of Anaesthesiology (ESA) guideline provides similar recommendations regarding TXA [15]. However, it advises against tourniquet use because of potential complications and limited effectiveness [16–18]. This aligns with the Dutch orthopaedic guideline knee arthroplasty, which also discourages tourniquet use in its latest update [19]. 

When comparing our study findings with these guidelines, most practices are in agreement, explaining the widespread use of TXA and the decrease in tourniquet use. The limited reporting of patient positioning to prevent venous congestion appears inconsistent with the guideline, though it is possible that surgeons do not perceive this as a blood-saving measure and therefore did not report it.

Regarding antiplatelet management, the Dutch guideline recommends discontinuing clopidogrel five days preoperatively and bridging with aspirin in high-bleeding-risk surgeries, while aspirin monotherapy can be continued [20]. This largely aligns with the ESA guideline which recommends discontinuation of clopidogrel 5 days before elective surgery and the discontinuation of aspirin if this is prescribed for primary prevention [15]. Most departments adhere to these recommendations although bridging with aspirin is less consistently practiced. DOACs and VKAs are appropriately discontinued preoperatively in all departments.

Postoperatively, European guidelines recommend a restrictive approach to drain use due to questionable efficacy in reducing blood loss [5, 15]. This is reflected in the decreased use of retransfusion drains and postoperative autologous blood transfusion observed in our study. In addition, the declining use of drains may be related to their impracticality within fast-track protocols, as they interfere with early mobilisation and patient comfort.

### Thromboprophylaxis

Most Dutch departments adhere to national thromboprophylaxis guidelines, recommending LMWH or DOAC for 28–35 days [21]. European guidelines similarly recommend LMWH or DOACs but recommend a shorter duration of 7 days, extendable up to 4 weeks in high VTE-risk individuals [5, 22]. For elective procedures with hospitalization under five days, thromboprophylaxis limited to the inpatient period may be considered.

### Strengths and limitations

Although the response rate was lower compared to previous surveys, we believe the results remain representative for Dutch practice. Responses were received from a diverse range of institutions, including university hospitals, teaching hospitals, general hospitals, and independent treatment centers, providing a representative overview of current practices across Dutch orthopedic departments. The survey included a wide range of blood-saving measures and thromboprophylaxis strategies, allowing for a complete overview of clinical practice. Furthermore, this enabled comparison with earlier surveys possible to demonstrate changes over time.

Common limitations of survey studies are a lack of control over respondents, inaccuracies in the responses and possible non-responder bias. The names of respondents are known which ensures that the questionnaire was completed by an orthopedic surgeon. Potential inaccuracies were reduced because in 35% of departments multiple orthopedic surgeons completed the questionnaire, allowing internal validation of the answers. Academic and teaching hospitals were overrepresented among responding departments, which is consistent with the larger department size and higher number of revision surgeries observed in the responder group. This may have resulted in a slight overestimation of practices that are more common in large hospitals or specialized centers which may follow recent guidelines more strictly than smaller centers. However, because all hospital types, including general hospitals and independent treatment centers, are represented among the responding departments, the overall sample still reflects the diversity of orthopedic practice settings. Moreover, the differences in procedure volumes and staffing between responders and non-responders were moderate rather than extreme. Therefore, although some degree of non-response bias cannot be completely excluded, it is unlikely that this has substantially affected the overall conclusions or the generalizability of the findings.

### Comparability between surveys

Although the questionnaire was designed to align with those used in previous surveys, changes in the availability of pharmacological agents (e.g., DOACs) and updates to clinical guidelines (e.g., recommendations regarding TXA) limit the possibility of directly comparing the use of specific pharmacological agents across survey periods. At the same time, progress in healthcare, such as minimal invasive surgery, the introduction of fast track surgery, optimized multimodel analgesia and short hospitalization following arthroplasty, directly led to the evolution of clinical guidelines. Consequently, the present results should be interpreted as a reflection of broader changes over time in blood management strategies and thromboprophylaxis practices, rather than as a direct comparison of individual pharmacological agents between surveys.

### Future perspectives and knowledge gaps

Blood-saving practices in Dutch arthroplasty care have evolved substantially since the first survey. The widespread adoption of blood-saving measures and their increased use in the early 2000 s reflected a growing awareness among surgeons of the importance of minimizing blood loss and wound complications. Subsequent improvements in surgical techniques, perioperative care, and early mobilisation programmes can be seen as an evolution of this awareness. The effectiveness of TXA in combination with fast track surgery, appears to have made many other blood saving measures unnecessary [23–25]. 

In contrast, the thromboprophylaxis guideline, especially in the Netherlands has remained largely unchanged from 2007 to 2023. This is remarkable as the venous thromboembolism (VTE) risk decreased from approximately 1.6% in 2007 to 0.9% in 2015, also partly driven by the introduction of fast-track surgery [26]. Other guidelines already suggest further shortening the duration of thromboprophylaxis or the use of acetylsalicylic acid to decrease the bleeding risk associated with LMWH and DOACs. However, a recent RCT showed an increased VTE incidence in patients using acetylsalicylic acid as thromboprophylaxis compared to LMWH [27]. Another option is further shortening of the duration of thromboprophylaxis. Multiple observational studies show in-hospital only thromboprophylaxis may be a safe option for low thrombosis risk individuals [28–30]. However, further research is needed to investigate in which patients thromboprophylaxis can be safely shortened.

## Conclusion

Concerns about perioperative blood loss appear to have become less prominent in routine arthroplasty practice. Advances in surgical techniques, improvements in postoperative care and the widespread use of tranexamic acid have likely contributed to this development and the subsequent change in clinical practice and institutional protocols. In contrast, thromboprophylaxis protocols have remained largely unchanged, apart from the introduction of DOACs. These findings highlight the opportunity to further balance the risks of bleeding and thrombosis following hip and knee arthroplasty.

## Supplementary Information

Below is the link to the electronic supplementary material.

Supplementary Material 1

## Data Availability

De-identified data will be made available for further research on reasonable request to the corresponding author.
