# Supplementary material for: 20 years of blood management and VTE prevention in orthopedic surgery in the Netherlands – a nationwide survey
Source: Arch Orthop Trauma Surg. 2026 Apr 23;146(1):157. doi: 10.1007/s00402-026-06301-8 (PMC13106243; doi:10.1007/s00402-026-06301-8)
Supplement: Supplementary file 1 — Supplementary Material 1 [file 402_2026_6301_MOESM1_ESM.docx]

20 years of blood management and VTE prevention in orthopedic surgery in the Netherlands – a nationwide survey

Supplementary material

|  | Intraoperative retransfusion | | |  | TXA |  | TXA IV | TXA Local | Normothermia | | Tourniquet | |
| --- | --- | --- | --- | --- | --- | --- | --- | --- | --- | --- | --- | --- |
|  | **2002** | **2007** | **2012** | **2023** | **2012** | **2023** | **2023** | **2023** | **2012** | **2023** | **2012** | **2023** |
| **THA** | 15% | 19% | 14% | 7% (CI 2% - 18%) | 14% | 97% (CI 87% - 99%) | 95% (CI 84% - 98%) | 12% (CI 5% - 24%) | 74% | 59% (CI 44% - 72%) | NA | NA |
| **RHA** | 40% | 54% | 56% | 15% (CI 8% - 28%) | 15% | 95% (CI 84% - 98%) | 92% (CI 81% - 97%) | 12% (CI 5% - 24%) | 73% | 59% (CI 44% - 72%) | NA | NA |
| **HHA** | 6% | 12% | 7% | 7% (CI 2% - 18%) | 8% | 88% (CI 76% - 95%) | 86% (CI 73% - 93%) | 11% (CI 5% - 23%) | 73% | 59% (CI 44% - 72%) | NA | NA |
| **TKA** | 10% | 14% | 9% | 7% (CI 2% - 18%) | 12% | 97% (CI 87% - 99%) | 95% (CI 84% - 98%) | 18% (CI 10% - 32%) | 72% | 59% (CI 44% - 72%) | 90% | 38% (CI 25% - 52%) |
| **RKA** | 16% | 24% | 26% | 8% (CI 3% - 19%) | 13% | 95% (CI 84% - 98%) | 92% (CI 81% - 97%) | 16% (CI 8% - 29%) | 71% | 59% (CI 44% - 72%) | 87% | 29% (CI 18% - 44%) |
| **UKA** | 6% | 11% | 8% | 7% (CI 2% - 18%) | 9% | 95% (CI 84% - 98%) | 92% (CI 81% - 97%) | 18% (CI 10% - 32%) | 73% | 59% (CI 44% - 72%) | 85% | 41% (CI 28% - 56%) |

Supplement 1 (part a): perioperative blood saving measures.*Patient positioning to prevent venous congestion. THA = Total hip arthroplasty, RHA = Revision hip arthroplasty, HHA = Hemi hip arthroplasty, TKA = Total knee arthroplasty, RKA = Revision knee arthroplasty, UKA = Unicondylar knee arthroplasty, TXA = Tranexamic acid. CIs represent 95% Confidence Intervals.

|  | Fibrin gel |  | Platelet gel | | Epinephrine | | Fibrin glue | Normovolemic  haemodilution | | Controlled hypotension | | Positioning* |
| --- | --- | --- | --- | --- | --- | --- | --- | --- | --- | --- | --- | --- |
|  | **2021** | **2023** | **2012** | **2023** | **2012** | **2023** | **2023** | **2012** | **2023** | **2012** | **2023** | **2023** |
| **THA** | 0% | 0% (CI 0% - 8%) | 0% | 0% (CI 0% - 8%) | 3% | 0% (CI 0% - 8%) | 4% (CI 1% - 15%) | 12% | 12% (CI 5% - 24%) | 11% | 10% (CI 4% - 22%) | 4% (CI 1% - 15%) |
| **RHA** | 0% | 0% (CI 0% - 8%) | 0% | 0% (CI 0% - 8%) | 2% | 0% (CI 0% - 8%) | 0% (CI 0% - 8%) | 15% | 12% (CI 5% - 24%) | 14% | 10% (CI 4% - 22%) | 4% (CI 1% - 15%) |
| **HHA** | 0% | 0% (CI 0% - 8%) | 0% | 0% (CI 0% - 8%) | 2% | 0% (CI 0% - 8%) | 0% (CI 0% - 8%) | 11% | 12% (CI 5% - 24%) | 12% | 10% (CI 4% - 22%) | 4% (CI 1% - 15%) |
| **TKA** | 1% | 0% (CI 0% - 8%) | 1% | 0% (CI 0% - 8%) | 5% | 3% (CI 1% - 13%) | 0% (CI 0% - 8%) | 12% | 12% (CI 5% - 24%) | 12% | 10% (CI 4% - 22%) | 5% (CI 2% - 16%) |
| **RKA** | 1% | 0% (CI 0% - 8%) | 1% | 0% (CI 0% - 8%) | 2% | 3% (CI 1% - 13%) | 0% (CI 0% - 8%) | 13% | 12% (CI 5% - 24%) | 13% | 10% (CI 4% - 22%) | 4% (CI 1% - 15%) |
| **UKA** | 1% | 0% (CI 0% - 8%) | 1% | 0% (CI 0% - 8%) | 3% | 3% (CI 1% - 13%) | 0% (CI 0% - 8%) | 11% | 12% (CI 5% - 24%) | 11% | 10% (CI 4% - 22%) | 4% (CI 1% - 15%) |

Supplement 1 (part b): perioperative blood saving measures.*Patient positioning to prevent venous congestion. THA = Total hip arthroplasty, RHA = Revision hip arthroplasty, HHA = Hemi hip arthroplasty, TKA = Total knee arthroplasty, RKA = Revision knee arthroplasty, UKA = Unicondylar knee arthroplasty, TXA = Tranexamic acid. CIs represent 95% Confidence Intervals.

|  | LMWH |  |  | LMWH + VKA* | |  | Aspirin | Fondaparinux | | DOAC | LMWH + DOAC | DOAC + Aspirin |
| --- | --- | --- | --- | --- | --- | --- | --- | --- | --- | --- | --- | --- |
|  | **2002** | **2007** | **2023** | **2002** | **2007** | **2023** | **2002** | **2007** | **2023** | **2023** | **2023** | **2023** |
| **THA** | 19% | 79% | 71% | 80% | 7% | 0% | 1% | 14% | 0% | 21% | 6% | 1% |
| **RHA** | 18% | 80% | 75% | 81% | 7% | 0% | 1% | 13% | 0% | 17% | 7% | 1% |
| **HHA** | 24% | 81% | 83% | 75% | 9% | 0% | NA | 10% | 0% | 9% | 7% | 1% |
| **TKA** | 22% | 80% | 73% | 77% | 8% | 0% | 1% | 14% | 0% | 21% | 6% | 1% |
| **RKA** | 20% | 78% | 77% | 79% | 7% | 0% | 1% | 13% | 0% | 16% | 7% | 1% |
| **UKA** | 17% | 79% | 71% | 82% | 7% | 0% | 1% | 12% | 0% | 21% | 7% | 1% |

Supplement 2: thromboprophylaxis strategies. THA = Total hip arthroplasty, RHA = Revision hip arthroplasty, HHA = Hemi hip arthroplasty, TKA = Total knee arthroplasty, RKA = Revision knee arthroplasty, UKA = Unicondylar knee arthroplasty, LMWH = low-molecular-weight-heparin, VKA = Vitamin K antagonist, DOAC = direct oral anticoagulant. Percentages may not total 100% due to rounding. *LMWH until adequate INR was reached

| 2023 | **14 days** | **21 days** | **28 days** | **30 days** | **35 days** | **42 days** |
| --- | --- | --- | --- | --- | --- | --- |
| **THA** | 0% | 0% | 49% | 18% | 24% | 9% |
| **RHA** | 0% | 0% | 50% | 17% | 22% | 11% |
| **HHA** | 0% | 0% | 50% | 17% | 19% | 14% |
| **TKA** | 11% | 1% | 49% | 13% | 15% | 9% |
| **RKA** | 8% | 1% | 47% | 13% | 17% | 14% |
| **UKA** | 12% | 1% | 47% | 13% | 17% | 10% |

Supplement 3: duration of postoperative thromboprophylaxis in 2023. THA

= Total hip arthroplasty, RHA = Revision hip arthroplasty, HHA = Hemi hip arthroplasty, TKA = Total knee arthroplasty, RKA = Revision knee arthroplasty, UKA = Unicondylar knee arthroplasty. Percentages may not total 100% due to rounding.

Supplement 4: Full study questionnaire.

Welkom bij deze survey over tromboseprofylaxe en bloedsparende maatregelen bij grote orthopedische ingrepen.

De totale tijd van deze survey bedraagt ongeveer 10 minuten.

U kunt deze survey zowel op de computer als op uw telefoon invullen.

Hartelijk dank dat u namens uw vakgroep de tijd wil nemen om deze survey in te vullen. Naam:

In welke ziekenhuizen/locaties is uw maatschap operatief werkzaam?

Hoeveel orthopedisch chirurgen zijn werkzaam bij uw vakgroep/maatschap?

| Aantal |
| --- |
| Stafleden |
| Chef de clinique |
| Fellows |

Uw orthopedische praktijk is:


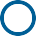
 Academisch
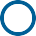
 Perifeer


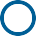
 Zelfstandig behandelcentrum

Heeft uw praktijk een overwegend uniform beleid voor tromboseprofylaxe?


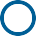

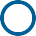
 Ja Nee

Heeft uw praktijk een overwegend uniform beleid voor bloedbesparende maatregelen?


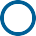
 Ja
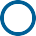
 Nee

De volgende vragen gaan over de tromboseprofylaxe die **routinematig** wordt toegepast bij de onderstaande operaties aan de **heup**:

THP (totale heupprothese) KHP (kophalsprothese) RevHP (revisie heupprothese)

PF# (proximale femurfractuur osteosynthese, incl. collum fracturen)

Gelieve aanvinken bij welke ingrepen er routinematig gebruik wordt gemaakt van: (Indien niet toegepast, graag vakje leeg laten)

Alle grote


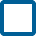

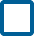

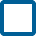

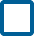

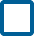

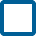

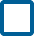

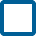

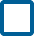

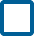

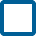

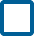

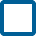

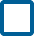

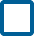

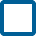

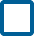

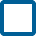

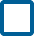

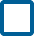

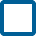

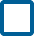

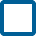

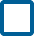

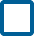

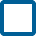

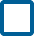

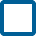

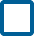

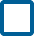

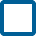

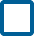

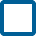

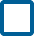

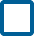

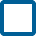

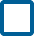

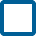

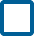
heupoperaties THP KHP RevHP PF#


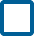


| LMW Heparine (b.v. Fraxiparine) |
| --- |
| UF Heparine |
| Coumarines (b.v. Sintrom) |
| Fondaparinux (Arixta) |
| Apixaban (Eliquis) |
| Dabigatran (Pradexa) |
| Edoxaban (Lixiana) |
| Rivaroxaban (Xarelto) |
| Acetylsalicylzuur |
| Gegradueerde elastische compressie |
| Intermitterende pneumatische compressie |
| Geen tromboseprofylaxe |
| Anders |


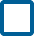

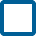

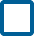

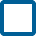

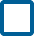

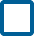

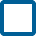

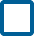

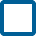

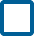

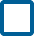

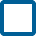

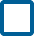

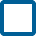

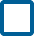

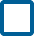

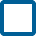

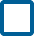

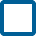

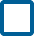

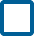

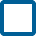

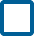

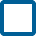

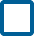
Indien andere profylaxe gebruikt wordt, welke profylaxe en op welke indicatie?

Bij welke operaties wordt de tromboseprofylaxe voortgezet na ontslag?

Gelieve het aantal dagen dat de profylaxe wordt voortgezet in het betreffende vakje invullen.

| Alle grote heupoperaties | THP | KHP | RevHP | PF# |
| --- | --- | --- | --- | --- |
| LMW Heparine (b.v. Fraxiparine) |  |  |  |  |
| UF Heparine |  |  |  |  |
| Coumarines (b.v. Sintrom) |  |  |  |  |
| Fondaparinux (Arixta) |  |  |  |  |
| Apixaban (Eliquis) |  |  |  |  |
| Dabigatran (Pradexa) |  |  |  |  |
| Edoxaban (Lixiana) |  |  |  |  |
| Rivaroxaban (Xarelto) |  |  |  |  |
| Acetylsalicylzuur |  |  |  |  |
| Gegradueerde elastische compressie |  |  |  |  |
| Intermitterende pneumatische compressie |  |  |  |  |
| Andere profylaxe |  |  |  |  |

Pagina 1 van 3

→

De volgende vragen gaan over de tromboseprofylaxe die **routinematig** wordt toegepast bij de onderstaande operaties aan de **knie**:

TKP (totale knieprothese) hKP (hemiknieprothese) RevKP (revisie knieprothese)

PT# (proximale tibiafractuur osteosynthese)

Gelieve aanvinken bij welke ingrepen er routinematig gebruik wordt gemaakt van: (Indien niet toegepast, graag vakje leeg laten)

Alle grote


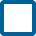

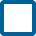

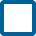

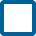

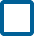

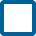

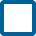

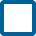

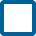

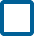

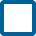

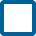

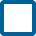

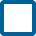

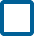

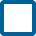

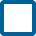

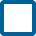

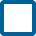

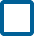

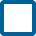

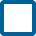

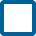

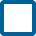

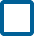

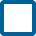

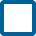

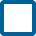
knieoperaties TKP hKP RevKP PT#

| LMW Heparine (b.v. Fraxiparine) |
| --- |
| UF Heparine |
| Coumarines (b.v. Sintrom) |
| Fondaparinux (Arixta) |
| Apixaban (Eliquis) |
| Dabigatran (Pradexa) |
| Edoxaban (Lixiana) |
| Rivaroxaban (Xarelto) |
| Acetylsalicylzuur |
| Gegradueerde elastische compressie |
| Intermitterende pneumatische compressie |
| Geen tromboseprofylaxe |
| Anders |

Indien andere profylaxe gebruikt wordt, welke profylaxe en op welke indicatie?

Bij welke operaties wordt de tromboseprofylaxe voortgezet na ontslag?

Gelieve het aantal dagen dat de profylaxe wordt voortgezet in het betreffende vakje invullen.

| Alle grote knieoperaties | TKP | hKP | RevKP | PT# |
| --- | --- | --- | --- | --- |
| LMW Heparine (b.v. Fraxiparine) |  |  |  |  |
| UF Heparine |  |  |  |  |
| Coumarines (b.v. Sintrom) |  |  |  |  |
| Fondaparinux (Arixta) |  |  |  |  |
| Apixaban (Eliquis) |  |  |  |  |
| Dabigatran (Pradexa) |  |  |  |  |
| Edoxaban (Lixiana) |  |  |  |  |
| Rivaroxaban (Xarelto) |  |  |  |  |
| Acetylsalicylzuur |  |  |  |  |
| Gegradueerde elastische compressie |  |  |  |  |
| Intermitterende pneumatische compressie |  |  |  |  |
| Andere profylaxe |  |  |  |  |

Bij welke van de volgende ingrepen wordt routinematig tromboseprofylaxe toegepast? Welke profylaxe wordt dan gebruikt?

(Indien geen profylaxe wordt toegepast, vakje leeg laten)

| Welke profylaxe wordt toegepast? |
| --- |
| Arthroscopie in dagbehandeling |
|  |
| Voorste kruisbandreconstructie |
|  |
| Achterste kruisbandreconstructie |
|  |
| Overige patiënten dagbehandeling |
|  |
| Onderbeensgipsen |
|  |
| Bovenbeensgipsen |

Indien alleen toegepast bij risicofactoren aanwezig, welke risicofactoren?

Indien er gebruikt gemaakt wordt van een LMW heparine, dan wordt gebruik gemaakt van:

Nadroparine (Fraxiparine®) Dalteparine (Fragmin®)

Enoxaparine (Inhixa®) Tinzaparine (Innohep®) n.v.t.

Op welk moment wordt er in de meeste gevallen gestart met LMW heparine?

>12 uur preoperatief

Perioperatief (4 uur voor tot 6 uur na) 6-12 uur postoperatief

12-24 uur postoperatief Anders

n.v.t.

Pagina 2 van 3

→

# Bloedsparende maatregelen

De volgende vragen gaan over bloedsparende maatregelen die routinematig pre-, peri-en post-operatief worden genomen om het verlies van bloed en stollingsproducten te beperken bij de volgende heup- of knie prothesiologie:

THP (totale heupprothese) KHP (kophalsprothese) RevHP (revisie heupprothese) TKP(totale knieprothese)

hKP (hemiknieprothese) RevKP (revisie knieprothese)

Worden de volgende anticoagulantia preoperatief gestaakt? Zo ja, hoe lang? (Indien niet gestaakt, vakje leeg laten)

| Hoeveel dagen voor de operatie? |
| --- |
| DOAC's (b.v.  Rivaroxaban) |
| NSAID's |
| Cox-2 Selectieve NSAID's |
| Clopidogrel |
|  |
| Ticagrelor |
|  |
| Acetylsalicylzuur |
|  |
| Dipyridamol |
|  |
| Acenocoumarol |
|  |
| Fenprocioumon |

Indien Clopidogrel wordt gestaakt, wordt dan er dan een andere trombocytenaggregatieremmer gestart? (b.v. Ascal)

Ja Nee

Clopidogrel wordt niet gestaakt

Bij welke operaties worden de volgende preoperatieve maatregelen routinematig genomen?

(Indien niet toegepast, graag vakje leeg laten)

Alle grote

operaties THP KHP RevHP TKP hKP RevKP

| Erythropoietine (EPO) |
| --- |
| Pre-op. autologe bloedtransfusie |
| Ijzer supplementatie (oraal) |
| Ijzer supplementatie (IV) |
| Anders |

Indien er andere maatregelen worden genomen, welke:

Indien een van deze preoperatieve maatregelen wordt gebruikt, welke Hb-waarde is de behandeldrempel ?

Bij welke operaties worden de volgende perioperatieve maatregelen routinematig genomen?

(Indien niet toegepast, graag vakje leeg laten)

Alle grote

operaties THP KHP RevHP TKP hKP RevKP

| Tranexaminezuur (Cyklokapron ®) (IV) |
| --- |
| Tranexaminezuur (Cyklokapron ®) (lokaal) |
| Intraoperatieve re-transfusie via cellsaver (Sangvia®) |
| Normothermie |
| Bloedleegte |
| Fibrine gel |

Alle grote

operaties THP KHP RevHP TKP hKP RevKP

| Platelet gel |
| --- |
| Epinephrine injecties |
| Fibrinelijm |
| Normovolemische hemodilutie |
| Gecontroleerde hypotensie |
| Positionering van de patiënt (voorkomen veneuze stuwing operatiegebied) |
| Anders |

Als er tranexaminezuur (Cyklokapron®) wordt toegepast, in welke dosering en op welk moment?

(Indien niet toegepast, graag vakje leeg laten)

| In welke dosering? | Wat is het moment van toediening? |
| --- | --- |
| Tranexaminezuur (Cyklokapron ®) (IV) |  |
| Tranexaminezuur (Cyklokapron ®) (lokaal) |  |

Bij welke operaties worden de volgende maatregelen routinematig genomen? (Indien niet toegepast, graag vakje leeg laten)

Alle grote

operaties THP KHP RevHP TKP hKP RevKP

| Postop. autologe transfusie |
| --- |
| Aanleggen van een drain |
| Compressie verband |
| Cryotherapie |
| Been elevatie |
| Anders |

Indien er andere maatregelen worden genomen, welke:

Wordt er in uw ziekenhuis gebruik gemaakt van 4-5-6 regel of een andere transfusiedrempel?

Dit is het einde van deze survey over tromboseprofylaxe en bloedsparende maatregelen binnen de orthopedie.

Wij danken u voor uw medewerking.

Druk op de knop rechtsonder om de survey af te sluiten.

→
